# Puerperal Septicæmia

**Published:** 1893-09

**Authors:** 


PUERPERAL SEPTICÆMIA—*Treatment of*—In the course of an important debate on this subject at the Glasgow Medico-Chirurgical Society, on February 3, 1893, Dr. Halliday Croom said:—In presence of a well-marked case of puerperal fever, one was often practically helpless, so that one's energies should be directed in the way of prevention. The wonderful diminution in the number of cases of puerperal fever was due to the introduction of antiseptics. In proof of this, Dr. Croom quoted from statistics published for Paris, Copenhagen, and Prague, and also referred to the experience of his own service in the Maternity Hospital in Edinburgh, where antiseptics had been at one time carried to the extent of delivering under the spray. After the introduction of antiseptics, he had had six years without a single septic death. In the larger hospitals there were always occurring little septic attacks, but the genuine wound-fever had almost disappeared. Another influence in the prevention of puerperal fever he considered to be the forceps. Delayed labor was one cause of puerperal fever. His rule, in hospital as well as private practice, was to allow no multipara to be in the second stage for more than three hours,and no primipara longer than four hours. He supposed he used forceps in over 70 per cent. of his cases. Supposing sepsis to have begun, there were one or two points which would be even then found useful in treatment. If septicæmia were taking place from retained membranes which had become septic, antisepticism might here also do wonders. The patient's temperature and pulse might have risen, and the abdomen become tender, but if one, with or without dilatation, washed out the uterine cavity with antiseptic fluid, the result would be very striking. In America, and on the Continent, some thought the curette an important addition to the treatment for retained membranes (even at full term), but he had not himself, while using it after abortion, employed it in such circumstances. The rule of those who advocated it was to wash out the vagina, and, if that were not sufficient, to wash out the uterus, and if even that failed to bring about improvement, to curette. Dr. Croom's feeling was that their hope as obstetricians lay in the use of antiseptics, and, secondly, in the early and careful use of the forceps.—*The Glasgow Medical Journal*, (*Braithwaite's Retıospect.*)

